# Beyond the physician shortage: infrastructure as a rate-limiting step in breast cancer care

**DOI:** 10.3389/fonc.2026.1826458

**Published:** 2026-07-07

**Authors:** Elio R. Bitar, Max O. Meneveau, Kaelyn C. Cummins, Olivia Sears, Mohamad El Moheb, Chengli Shen, Susan Kim, Mackenzie M. Mayhew, Samantha M. Ruff, Allan Tsung

**Affiliations:** Department of Surgery, University of Virginia School of Medicine, Charlottesville, VA, United States

**Keywords:** breast cancer, geographic variation, health disparities, healthcare infrastructure, provider density, screening access

## Abstract

**Background:**

Breast cancer is the most common cancer among women in the United States, yet early detection and survival vary widely across regions. Physician shortages and limited facility access are both implicated, but whether local infrastructure modifies the association between physician workforce availability and breast cancer outcomes remains uncertain. Clarifying this relationship is critical to guide policy and investment.

**Study design:**

This county-level cross-sectional study used data from the Area Health Resource File, Behavioral Risk Factor Surveillance System, and Centers for Disease Control and Prevention. Provider density was defined as the number of primary care physicians and obstetrician-gynecologists (screening) or surgeons and radiation oncologists (treatment) per 100, 000 women aged 50–74. Facility availability was defined as the presence of mammography services (screening) and surgical and chemotherapy services (treatment). A neighboring-county facility index estimated regional access. Outcomes were county-level screening rates, late-stage diagnoses, and mortality. Adjusted generalized linear models with provider density-by-facility availability interaction terms estimated whether the association between provider density and breast cancer outcomes in women aged 50–74 differed according to local facility availability.

**Results:**

High-density counties were more likely to be urban, socioeconomically advantaged, and have mammography facilities (p<0.001). In adjusted models, greater provider density was associated with increased screening (β=1.49, p<0.001) and decreased late-stage diagnosis (β=−1.29, p<0.001) and mortality (β=−1.09, p<0.001), but only when facilities were available. Among counties lacking local facilities, more neighboring counties with screening or treatment resources was associated with higher screening (β=1.23, p=0.047) and lower mortality (β=−1.69, p=0.023).

**Conclusions:**

Among women aged 50–74 years, provider density was associated with more favorable outcomes only when facilities were available, suggesting that increasing workforce availability alone may be insufficient without local healthcare infrastructure. Aligning workforce expansion with infrastructure investment may reduce disparities in breast cancer outcomes.

## Introduction

Breast cancer remains the most commonly diagnosed malignancy among women in the United States (U.S.) and is a leading cause of cancer-related death (1, 2). Despite advances in screening and treatment, significant geographic and sociodemographic disparities persist (3, 4). Early detection through mammography is a cornerstone of improving survival, yet access to screening resources is unevenly distributed, with prior studies showing that over one-quarter of U.S. counties have no local mammography facilities (5–8). At the same time, shortages in primary care physicians, radiologists, surgeons, and oncologists may further limit timely screening, diagnosis, and treatment. However, the relative importance of facility availability and provider density, and whether local facility infrastructure modifies the association between provider density and breast cancer outcomes remains poorly understood.

With the growing physician shortage, disparities in access to care are expected to widen, and increased attention is being directed to the impact of workforce capacity on cancer outcomes (9). Provider density is an increasingly important determinant of cancer outcomes across many domains of oncology (10–12), and areas with lower density of primary care physicians and specialists consistently show decreased screening rates and worse overall outcomes in cutaneous melanoma (11), hepatobiliary tumors (12), and colorectal cancer (10). However, relatively little is known about how breast cancer–specific provider availability influences outcomes across different points in the care continuum. In particular, the relationship between screening provider density, mammography facility availability, and screening uptake remains unclear. Similarly, the combined influence of breast cancer treatment provider density and treatment facility availability on late-stage diagnosis and mortality has not been well characterized.

Understanding whether barriers to screening and treatment arise primarily from physician workforce or healthcare infrastructure is essential for guiding policies to reduce breast cancer disparities. Yet, no national study has comprehensively evaluated these factors together at the county level. To address this gap, we examined whether mammography facility availability modifies the association between county-level screening provider density and screening rates and late-stage breast cancer diagnosis in women aged 50–74 years. We further examined whether treatment facility availability modifies the association between treating provider density and breast cancer mortality, providing a comprehensive evaluation of how workforce availability relates to outcomes across the breast cancer care continuum in the context of local healthcare infrastructure.

## Methods

### Data source

The 2023–2024 Area Health Resource File (AHRF), maintained by the Health Resources and Services Administration (HRSA), was used to obtain county-level provider, facility, demographic, socioeconomic, and geographic data. The AHRF provides data on providers, facilities, demographics, and hospital utilization and expenditures (13). Specifically, AHRF was used to obtain provider counts by specialty, medical and surgical facilities availability (mammography, operating rooms, chemotherapy), population denominators by age group, county demographic characteristics, median household income, race and ethnicity distribution, proportion of residents aged ≥65 years, and urban-rural classification. County-level screening data and other health measures were obtained from the Centers for Disease Control and Prevention (CDC) Population Level Analysis and Community Estimates (PLACES) (14) platform, which reports model-based local estimates collected by the 2023 Behavioral Risk Factor Surveillance System (BRFSS). The BRFSS is a nationally representative telephone survey of noninstitutionalized U.S. adults that collects information on health-related behaviors and chronic medical conditions (15). PLACES/BRFSS were used to obtain mammography screening rates, insurance coverage, and transportation-related measures. County-level breast cancer incidence, late-stage diagnosis, and mortality data were obtained from the CDC Wide-ranging Online Data for Epidemiologic Research (WONDER), a publicly accessible platform that enables users to query and analyze data on births, deaths, cancer, tuberculosis, vaccinations, and other health indicators. County Federal Information Processing Standards (FIPS) codes were used to merge data across these sources.

### Dependent and independent variables

The primary independent variable was provider density, stratified into tertiles. Provider density was categorized into tertiles to provide an interpretable comparison of counties with low, moderate, and high workforce availability, while avoiding assumptions of a strictly linear association between provider density and outcomes. Tertiles also provided adequate numbers of counties within each provider-density stratum when further stratified by facility availability, allowing evaluation of the joint contribution of workforce and infrastructure availability. Provider-density measures were constructed using specialty-specific workforce variables available in the AHRF. For screening analyses, screening provider density was defined as the number of primary care physicians (PCPs) and obstetrician-gynecologists (OB-GYNs) per 100, 000 women aged 50–74 years. For mortality analyses, breast cancer treatment provider density was defined as the number of general surgeons and radiation oncologists per 100, 000 women aged 50–74 years. These terms are used consistently throughout the manuscript to distinguish provider availability relevant to screening from provider availability relevant to treatment. Facility availability measures were similarly based on AHRF-available county-level infrastructure variables. Screening facility availability was defined as the presence of at least one facility offering full-field digital mammography (FFDM) or any other accredited mammography facility within a county. Treatment resource availability was defined as the presence of both surgical and chemotherapy facilities within the county.

To further capture regional access among counties lacking local facilities, we created a neighboring-county facility index. Among counties lacking local facilities, counties were not assigned a value of 0 for facility access. For each county without a local facility, each contiguous neighboring county was coded as 1 if the relevant facility was present and 0 if absent. The index was then calculated as the sum of neighboring counties with the facility divided by the total number of contiguous neighboring counties. Thus, the index ranged from 0 to 1, where 0 indicated that none of the adjacent counties had the facility and 1 indicated that all adjacent counties had the facility. For example, if a county had four contiguous neighboring counties and all four had the relevant facility, the index value was 1.00; if two of four had the facility, the index value was 0.50. For screening analyses, the index represented neighboring mammography facility availability. For mortality analyses, the treatment facility index represented neighboring availability of both operating room and chemotherapy facilities. Other covariates included the county-level proportion of uninsured individuals, median income, urban-rural status, proportion of the population aged ≥65, and additional sociodemographic and health-quality indicators.

Primary outcomes were breast cancer screening rates, stage at diagnosis, and mortality. Late-stage diagnosis was defined as regional or distant metastasis at presentation. Together, these outcomes captured the continuum of breast cancer control, from early detection to treatment administration and survival.

### Statistical analysis

Categorical variables were summarized as frequencies and percentages [N (%)], while continuous variables, after confirming non-normality using the Shapiro-Wilk test, were reported as medians with interquartile ranges (IQR). For unadjusted comparisons, the Wilcoxon rank-sum test was used for two-group comparisons and the Kruskal-Wallis test was used for comparisons across three provider-density tertiles. Categorical variables were compared using Chi-square or Fisher’s exact tests, as appropriate. Generalized linear models (identity link; conceptual model structure shown in **Supplementary Figure 1**) were employed to estimate absolute differences (β coefficients) in screening, late-stage incidence, and mortality. All models were adjusted for county-level age distribution, racial and ethnic composition, median household income, insurance coverage, rural-urban status, and lack of reliable transportation. Models evaluating late-stage breast cancer diagnosis were additionally adjusted for county-level mammography screening rate to evaluate whether provider density exerted effects independent of screening uptake, while models evaluating breast cancer mortality were additionally adjusted for county-level late-stage breast cancer diagnosis rate. For screening analyses, an interaction term between provider density and mammography facility availability tested whether associations differed by the presence of local screening infrastructure. For mortality, interaction terms assessed effect modification between provider density and treatment facility availability. Interaction effects were evaluated using stratified models and marginal means. Estimates are reported as β coefficients with corresponding 95% confidence intervals (CI), and statistical significance was set at *p ≤* 0.05. As a sensitivity analysis, provider density was also modeled as a continuous variable, including an interaction term with mammography facility availability, to assess whether the primary tertile-based findings were robust to modeling provider density continuously. In an additional sensitivity analysis, models were stratified by facility availability, with low provider density used as the reference group within each facility-availability stratum. Statistical analyses were conducted using the IBM Statistical Package for Social Sciences (SPSS) software version 29, and R software version 4.5.1. This study was conducted and reported in accordance with the STROBE statement.

## Results

### County-level demographic, socioeconomic and population health characteristics

**Figure 1:**
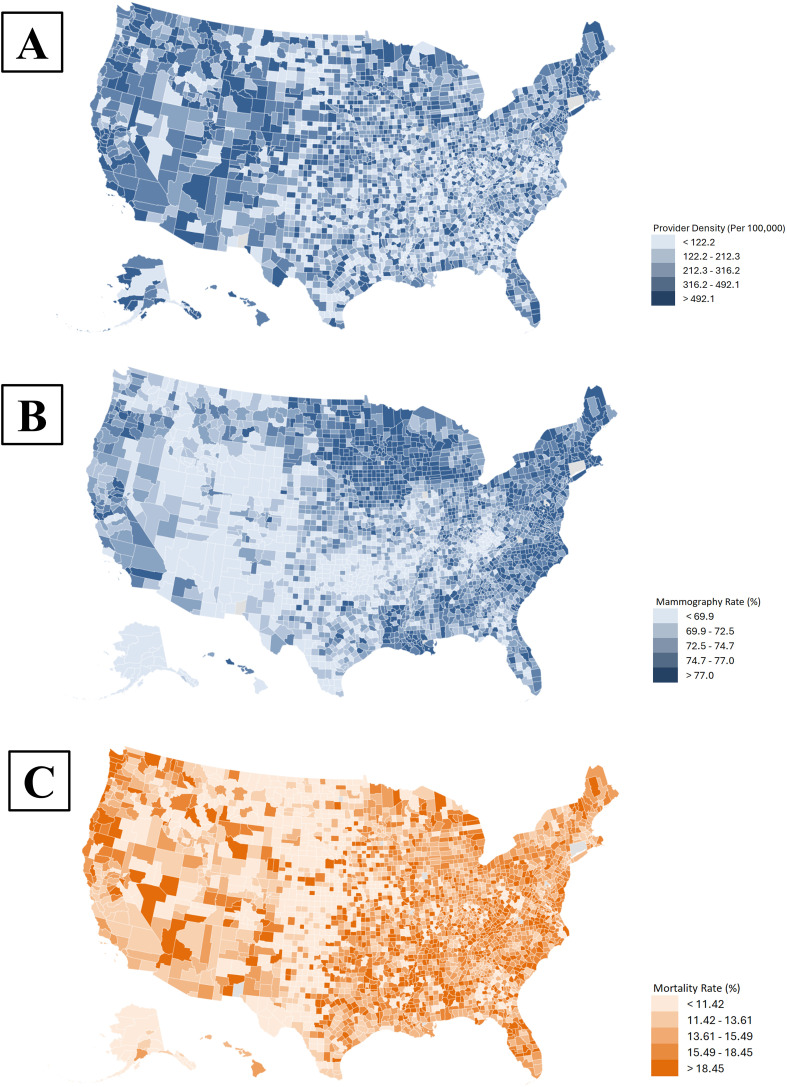
**(A)** Screening provider density per 100, 000 women aged 50–74 across U.S. Counties. **(B)** Mammography Rates (Percent of Women Aged 50–74 Who Underwent Mammography Screening) Across U.S. Counties. **(C)** Breast Cancer-Specific Mortality Rates Across U.S. Counties. Sequential palettes were used, with blue shades representing provider-resource measures and orange shades representing mortality as a disease-burden measure. Darker shades indicate higher county-level values within each panel.

**Table 1** summarizes characteristics of the 3, 108 included U.S. counties, 62.6% of which were rural and 37.4% urban. Median screening provider density overall was 263.5 per 100, 000 women aged 50–74, ranging from 105.5 (IQR 38.7–144.6) in low-density counties to 533.1 (IQR 429.6–701.8) in high-density counties (p<0.001). Geographic variation was evident, with lower-density counties concentrated in the South and Midwest, and higher-density counties in the Northeast and West Coast (**Figure 1A**).

**Table 1:** County-level demographic, socioeconomic and health system characteristics.

Variable % [median (IQR)]	Total (n=3, 108)	Screening provider density			P-value
		Low (n=1, 036)	Moderate (n=1, 036)	High (n=1, 036)	
Provider Density	263.5 (144.6-429.6)	105.5 (38.7-144.6)	263.8 (221.2-306.9)	533.1 (429.6-701.8)	<0.001
Female	49.9 (49.0-50.7)	49.6 (48.5-50.4)	49.9 (49.1-50.6)	50.2 (49.4-50.9)	<0.001
Race					
White	90.6 (78.8-94.9)	92.2 (78.0-95.5)	91.7 (82.0-95.1)	87.7 (75.7-93.5)	<0.001
Black	2.7 (1.0-10.8)	2.3 (0.9-11.3)	2.6 (1.0-9.0)	3.4 (1.2-11.6)	0.010
Asian	0.8 (0.5-1.6)	0.6 (0.4-0.9)	0.8 (0.6-1.2)	1.6 (0.9-3.6)	<0.001
Hispanic Ethnicity	5.1 (2.8-11.0)	4.1 (2.5-8.3)	5.0 (2.8-10.3)	8.5 (6.6-14.1)	<0.001
Median Household Income	60851.0 (52524.3-71494.8)	55822.0 (47866.0-64026.5)	60060.0 (52175.5-68863.0)	66000.0 (57564.0-77502.0)	<0.001
>65 years old	20.1 (17.4-23.2)	20.9 (18.6-23.4)	20.0 (17.8-23.3)	18.4 (15.8-22.0)	<0.001
Smoking	17.7 (15.5-20.4)	19.6 (17.2-22.0)	16.0 (18.0-20.4)	16.1 (13.7-18.2)	<0.001
Obese	38.4 (35.3-41.0)	39.9 (37.8-42.1)	39.4 (36.7-41.4)	36.7 (32.9-39.7)	<0.001
Depression	24.3 (21.9-26.5)	24.8 (22.3-27.2)	24.6 (22.1-26.9)	23.6 (21.1-25.8)	<0.001
Lack of insurance	10.5 (8.0-14.0)	12.9 (9.5-16.9)	11.3 (8.5-15.1)	9.7 (7.5-12.9)	<0.001
Sedentary Lifestyle	26.0 (22.8-29.8)	28.9 (25.5-31.8)	26.9 (23.7-30.3)	24.0 (20.8-27.4)	<0.001
Short sleep duration	37.7 (34.4-40.2)	38.9 (36.0-41.1)	37.8 (34.6-40.3)	35.6 (32.9-38.7)	<0.001
Cognitive Disability	16.1 (14.0-18.3)	17.6 (15.7-19.6)	16.4 (14.6-18.5)	14.8 (13.3-16.7)	<0.001
Any Disability	32.0 (28.3-36.5)	35.3 (31.5-38.8)	33.0 (29.3-37.3)	29.9 (26.9-33.8)	<0.001
Fair/Poor self-rated Health Status	18.9 (16.0-22.6)	21.5 (18.2-24.6)	19.4 (16.5-22.9)	17.0 (14.8-20.2)	<0.001
Frequent Physical Distress	13.5 (11.9-15.2)	14.7 (13.1-16.0)	13.7 (12.2-15.4)	12.4 (11.3-14.0)	<0.001
Frequent Mental Distress	18.6 (17.1-20.1)	19.4 (18.2-20.9)	18.9 (17.5-20.2)	16.4 (17.6-19.0)	<0.001
No Emotional Support	25.1 (22.8-28.3)	26.1 (23.5-29.3)	25.0 (22.9-27.7)	24.3 (22.2-27.4)	<0.001
Food Insecurity	15.1 (12.0-19.7)	17.3 (13.5-22.1)	15.2 (12.1-19.4)	13.3 (10.8-16.9)	<0.001
Housing Insecurity	13.2 (11.2-16.4)	14.5 (12.1-18.1)	13.3 (11.4-16.2)	12.3 (10.5-14.8)	<0.001
Social Isolation	34.2 (32.5-35.9)	34.7 (33.1-36.3)	34.2 (32.4-35.9)	33.6 (32.0-35.5)	<0.001
No Reliable Transportation	9.2 (7.8-11.2)	10.1 (8.5-12.3)	9.2 (7.9-11.1)	8.5 (7.3-10.0)	<0.001
Rurality					<0.001
Urban	1, 161 (37.4%)	359 (34.7%)	304 (29.3%)	498 (48.1%)	
Rural	1, 947 (62.6%)	677 (35.6%)	732 (70.7%)	538 (51.9%)	
At Least One Hospital With Mammography Facilities	1, 684 (53.7%)	303 (29.2%)	590 (56.9%)	784 (75.7%)	<0.001

Counties with higher screening provider density were more urban and socioeconomically advantaged. Median household income increased from $55, 822 to $66, 000 across density tertiles (p<0.001), while the uninsured rates declined from 12.9% to 9.7% (p<0.001). Low-density counties had a greater proportion of residents aged ≥65 years (20.9% vs. 18.4%, p<0.001).

Health behaviors and self-reported health metrics also varied. Residents of low-density counties were more likely to be smokers (19.6% vs. 16.1%), obese (39.9% vs. 36.7%), lead sedentary lifestyles (28.9% vs. 24.0%), and self-rate their health as poor or fair (21.5% vs. 17.0%) (all p<0.001). They also reported higher rates of depression (24.8% vs. 23.6%), frequent physical distress (14.7% vs. 12.4%), frequent mental distress (19.4% vs. 16.4%), disability (35.3% vs. 29.9%), and lack of emotional support (26.1% vs. 24.3%) (all p<0.001). Other indicators of health and social vulnerability were higher in low-density counties, including food insecurity (17.3% vs. 13.3%), housing insecurity (14.5% vs. 12.3%), and lack of reliable transportation (10.1% vs. 8.5%) (all p<0.001).

Importantly, healthcare infrastructure also differed substantially. Only 29.2% of low-density counties had at least one hospital with mammography facilities compared with 75.7% of high-density counties (p<0.001).

### Association between screening provider density and breast cancer screening

Mammography screening rates varied geographically, mirroring screening provider density. Lower screening rates were observed across rural counties in the South and Midwest and higher rates were seen in the Northeast and West Coast (**Figure 1B**). The median county-level mammography screening rate was 73.6% (IQR 70.6–76.4), increasing from 73.2% in low-density to 75.4% in high-density counties (Kruskal-Wallis p<0.001; **Table 2**).

**Table 2:** Unadjusted analysis of breast cancer screening, late-stage diagnosis, and mortality relative to provider density.

Outcome % [median (IQR)]	Total	Provider density			p-value
		Low	Moderate	High	
Screening Rates	73.60 [70.60-76.40]	73.20 [70.50-75.80]	73.90 [70.80-76.70]	75.40 [72.60-77.90]	**<0.001**
Late-stage Diagnosis	31.70 [28.90-35.40]	33.30 [29.80-37.80]	32.10 [29.30-35.70]	30.80 [28.30-33.80]	**<0.001**
Mortality Rate	14.46 [12.02-17.54]	16.14 [12.91-19.88]	14.49 [12.27-17.08]	13.68 [11.55-16.19]	**<0.001**

*Values are county-level percentages summarized as median (interquartile range). P-values were calculated using the Kruskal-Wallis test across provider-density tertiles.

P-values < 0.05 are bolded are were considered statistically significant.

**Figure 2:**
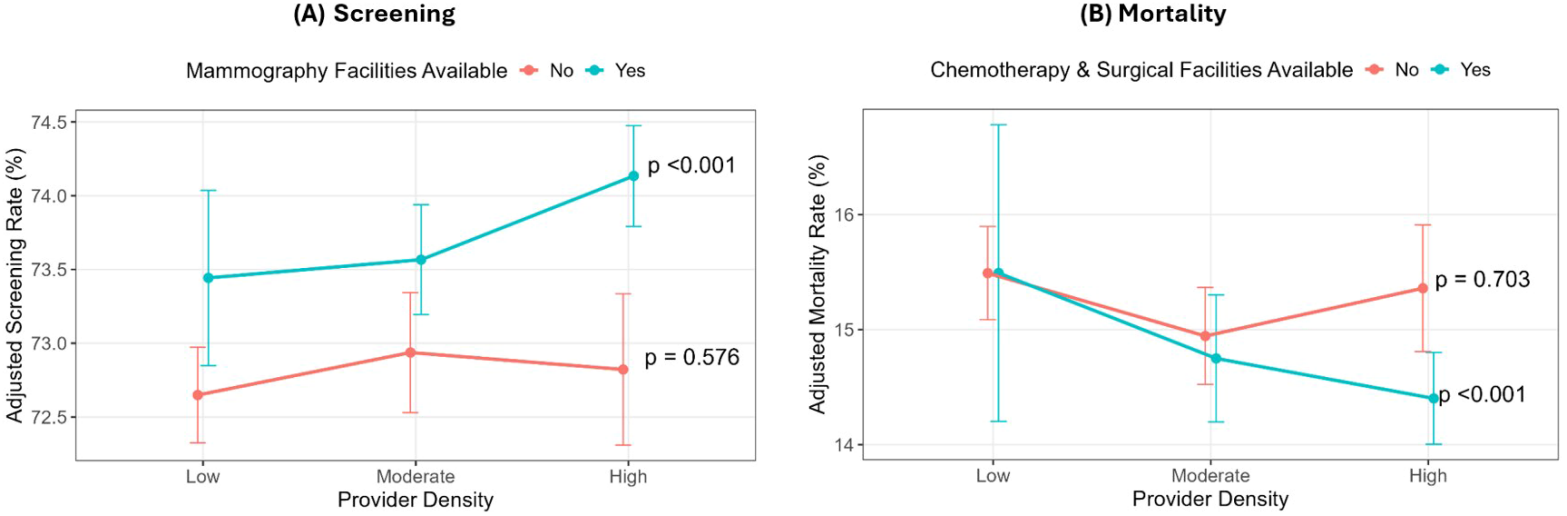
Adjusted breast cancer screening **(A)** and Mortality **(B)** Rates by Provider Density and Facility Availability.

On multivariable analysis (**Table 3**), the association with screening rates depended on the presence of local facilities. In counties without facilities, moderate and high screening provider density were not associated with higher screening rates compared with low provider density, with adjusted absolute differences of 0.29% (95% CI, −0.22 to 0.79; p=0.264) and 0.17% (95% CI, −0.43 to 0.78; p=0.576), respectively. In contrast, in counties with facilities, moderate density was associated with a 0.92% higher screening rate (95% CI 0.43–1.41, p<0.001), and high density with a 1.49% higher rate (95% CI 1.00–1.97, p<0.001), compared with low-density counties. Low-density counties with facilities also showed a 0.99% higher screening rate compared with their counterparts without facilities (95% CI 0.32–1.67, p=0.004). This effect modification is illustrated in **Figure 2A**. In a supplementary stratified sensitivity analysis using low provider density as the reference within each facility-availability stratum, provider density remained unassociated with screening rates among counties without mammography facilities, whereas high provider density was associated with higher screening rates among counties with mammography facilities, supporting the robustness of the observed effect modification. This finding was robust in a sensitivity analysis modeling screening provider density continuously, which similarly showed no significant association between provider density and screening rates in counties without mammography facilities, but a significant positive association in counties with mammography facilities (Supplementary Results). Among counties lacking local facilities, a higher ratio of adjacent counties with mammography facilities was associated with a 1.23% higher breast cancer screening rate (95% CI 0.02–2.44, p=0.047).

**Table 3:** Adjusted analysis of breast cancer screening, late-stage diagnosis, and mortality relative to provider density and facility availability.

Outcome	Facility availability	Provider density	Adjusted β	p-value
Screening Rate	Mammography Facilities Unavailable	Low (Ref)	0.00 (Reference)	–
		Moderate	0.29 (-0.22; 0.79)	0.264
		High	0.17 (-0.43; 0.78)	0.576
	Mammography Facilities Available	Low	0.994 (0.32; 1.67)	**0.004**
		Moderate	0.92 (0.43; 1.41)	**<0.001**
		High	1.49 (1.00; 1.97)	**<0.001**
Late-stage Diagnosis	–	Low (Ref)	0.00 (Reference)	–
		Moderate	-0.57 (-1.19; 0.05)	0.072
		High	-1.29 (-1.95; -0.63)	**<0.001**
Mortality	Treatment Facilities Unavailable	Low (Ref)	0.00 (Reference)	–
		Moderate	-0.55 (-1.12; 0.03)	0.063
		High	-0.13 (-0.81; 0.55)	0.703
	Treatment Facilities Available	Low	0.00 (-1.35; 1.35)	0.999
		Moderate	-0.742 (-1.44; -0.05)	**0.036**
		High	-1.09 (-1.68; -0.50)	**<0.001**

*All models were adjusted for county-level age distribution, racial and ethnic composition, median household income, insurance coverage, rural-urban status, and lack of reliable transportation. Models evaluating late-stage breast cancer diagnosis were additionally adjusted for county-level mammography screening rate, while models evaluating breast cancer mortality were additionally adjusted for late-stage breast cancer diagnosis rate.

P-values < 0.05 are bolded are were considered statistically significant.

### Association between screening provider density and late-stage breast cancer diagnosis

We next examined the association between screening provider density and late-stage diagnosis. Higher screening rates in high-density counties paralleled lower late-stage diagnoses. The median proportion of cases diagnosed at a late stage was 31.7% (IQR 28.9–35.4%), ranging from 33.3% in low-density to 30.8% in high-density counties (Kruskal-Wallis p<0.001; **Table 2**). In adjusted models adjusting for screening rates, compared with low-density counties, 0.57% lower proportion of late-stage diagnoses, although this difference was not statistically significant (95% CI, −1.19 to 0.05; p=0.072). High-density counties had a significantly lower proportion of late-stage diagnoses, with an adjusted absolute difference of −1.29% compared with low-density counties (95% CI, −1.95 to −0.63; p<0.001).

### Association between treating provider density, treatment facility availability, and mortality

Breast cancer mortality varied geographically, with higher rates in rural counties across the South and Midwest and lower rates in the Northeast and West Coast (**Figure 1C**). The median county-level mortality rate was 14.5% (IQR 12.0–17.5%), ranging from 16.1% in low-density to 13.7% in high-density counties (Kruskal-Wallis p<0.001; **Table 2**). On multivariate analysis adjusting for late-stage diagnoses (**Table 3**), the association with mortality differed by treatment facility presence. Among counties without treatment facilities, moderate and high treatment provider density were not significantly associated with mortality compared with low treatment provider density, with adjusted absolute differences of −0.55% (95% CI, −1.12 to 0.03; p=0.063) and −0.13% (95% CI, −0.81 to 0.55; p=0.703), respectively. In contrast, in counties with treatment facilities, moderate treatment provider density was associated with a 0.74% lower mortality rate compared with low treatment provider density (95% CI −1.44 to −0.05, p=0.036) and high treatment provider density was associated with a 1.09% lower rate (95% CI −1.68 to −0.50, p<0.001). This effect modification is illustrated in **Figure 2B**. In a stratified sensitivity analysis using low provider density as the reference within each facility-availability stratum, treatment provider density was not significantly associated with mortality among counties without treatment facilities, whereas both moderate and high provider density were associated with lower mortality among counties with treatment facilities (Supplementary Results). This association persisted in a sensitivity analysis modeling treatment provider density continuously, which similarly showed no significant association between provider density and mortality rates in counties without treatment facilities, but a significant positive association in counties with facilities (Supplementary Results). Among counties lacking local facilities, a higher ratio of adjacent counties with treatment facilities was associated with a 1.69% lower breast cancer mortality rate (95% CI, −3.14 to −0.23; p=0.023).

## Discussion

Breast cancer is a major healthcare burden in the U.S., with persistent geographic and sociodemographic disparities in screening and outcomes. While studies have explored the socioeconomic and geographic determinants of access to breast cancer care, the combined impact of physician workforce and healthcare infrastructure on outcomes is less understood. To our knowledge, this is the first national study to examine the interaction between provider density and facility availability in relation to breast cancer outcomes in women aged 50–74 years at the county level across the U.S. By stratifying outcomes by facility presence, our analysis provides a clearer understanding of how workforce capacity and infrastructure interact to influence screening, stage at diagnosis, and mortality, thereby offering insights to guide effective policy and investment strategies to improve breast cancer care.

Social determinants of health, particularly healthcare access and structural barriers to care, have been increasingly recognized as key drivers of disparities in cancer outcomes (16). Beyond individual-level factors such as insurance and socioeconomic status, structural elements of healthcare shape when and how patients access cancer screening, diagnosis, and treatment (17). Recent reports warn of a serious, growing U.S. physician shortage. The Association of American Medical Colleges projects a shortfall of up to 86, 000 physicians by 2036 (9), while the HRSA’s 2024 workforce report forecasts even larger gaps, reaching up to 187, 130 physicians by 2037, especially in nonmetropolitan areas (18). Thus, many programs have been implemented to recruit and retain physicians to rural areas (19). As such, provider density has emerged as an important structural factor influencing healthcare outcomes (20), including cancer outcomes such as melanoma (21, 22), colorectal cancer (12, 23), and hepatobiliary tumors (12).

In breast cancer, Ferrante et al. found that higher PCP density in Florida was associated with earlier breast cancer diagnosis (24). Another Ontario study by Gorey et al. found that five-year breast cancer survival rates were lower in areas where OBGYNs and PCPs were undersupplied (25). Because breast cancer treatment providers were not directly studied, the lower survival observed in provider-undersupplied areas may reflect lower screening uptake, delayed diagnosis, or other downstream access barriers rather than the direct effect of treatment provider availability.

Beyond the role of workforce density, the contribution of local mammography facility availability to breast cancer screening remains less clear, and prior regional studies have reported inconsistent findings. Coughlin et al. reported no association between the number of mammography facilities and mammography use (26). In contrast, Elkin et al. found that women in areas with inadequate mammography capacity were significantly less likely to receive breast cancer screening (27). Similarly, Elting et al. observed that women in regions with mammography facilities had higher odds of receiving screening, adjusting for socioeconomic and healthcare factors (7). Our national analysis of women aged 50–74 years shows that higher screening provider density is associated with greater mammography use, but only when mammography facilities are available. In our cohort, low- and high-density counties had median screening provider densities of 105.5 and 533.1 providers per 100, 000 women aged 50–75 years, respectively, corresponding to a difference of 427.6 providers per 100, 000 women. Based on our model estimates, in a hypothetical county with a population similar to New York (~250, 000 women aged 50–74), moving from low to high-provider density and having local screening facilities would be expected to yield approximately 3, 750 additional women undergoing mammography in this age group. This estimate is intended to illustrate the magnitude of the model-derived association in a large county and should not be interpreted as a projection applicable to all counties. Screening rates are also higher when more adjacent counties have screening facilities.

Apart from screening, the evidence also remains inconclusive as to whether facility availability influences stage at diagnosis. Dai et al. found that greater mammography accessibility negatively correlated with late-stage diagnosis, suggesting that improved facility availability may facilitate earlier detection (28). Similarly, Elting et al. reported that women living in counties with mammography facilities were less likely to present with advanced-stage disease (7). However, other analyses found no significant association between geographic access to mammography and stage at diagnosis, indicating that factors beyond geographic proximity may shape stage at presentation (29, 30). In our analysis, higher screening provider density was in fact associated with fewer late-stage diagnoses, highlighting the complementary role of workforce availability alongside facility access.

Mortality outcomes have been less frequently examined. Longacre et al. found that women living farther from radiation facilities were less likely to receive guideline-concordant radiation therapy, and had worse survival (31). Ward et al., using a microsimulation model of global breast cancer survival, estimated that scaling up access to imaging and treatment facilities could substantially improve 5-year survival (32). Taken together, these findings suggest that the impact of facility availability is not uniform, and breast cancer outcomes are likely shaped by interacting socioeconomic, geographic and workforce factors. In this context, our findings firmly establish that the mortality benefit of higher treating provider density is confined to counties with available surgical and chemotherapy facilities, emphasizing the necessity of both workforce and infrastructure. A county similar to New York, based on our estimates, would be expected to experience a reduction of more than 2, 700 breast cancer deaths annually with the availability of treatment facilities and an increase from low to high provider density. This estimate is intended to illustrate the magnitude of the model-derived association in a large county and should not be interpreted as a causal projection or as a change that would apply uniformly across all counties. We also found that a higher ratio of adjacent counties with treatment facilities is associated with lower mortality rates in counties lacking facilities.

Our results emphasize that workforce and infrastructure should not be viewed in isolation. While increasing the physician workforce has long been proposed as a solution for cancer care disparities, this would only be effective when accompanied by parallel investments in infrastructure. Expanding the number of physicians without adequate facilities would produce a workforce unable to deliver the necessary care, while building facilities without sufficient staffing would lead to underutilization and inefficiency. In order to address the physician shortage in rural communities, targeted incentives have been developed to support the establishment of residency programs (33–35). Building on the interdependence of workforce and infrastructure suggested by our findings, infrastructure development itself may indirectly contribute to workforce expansion. Hospitals with robust medical and surgical facilities are more likely to host residency and fellowship programs, thereby increasing local provider density over time. Strengthening facility capacity in underserved regions could thus not only improve immediate access to cancer care, but also create a sustainable pipeline of physicians who are likely to remain and practice in those areas.

Our study has several limitations. First, its ecological nature implies that the associations we observed may not necessarily reflect individual-level relationships. Clinicopathologic variables such as tumor biology, detailed staging, comorbidities, and treatment adherence were not included. However, because this study was designed as a county-level analysis examining associations between healthcare infrastructure and population outcomes, incorporation of individual patient-level characteristics would not be applicable. These variables are inherently patient-specific and cannot be directly integrated into population-level resource analyses; therefore, their absence should be interpreted within the context of the study’s system-level focus. Second, AHRF measures may not fully capture real-time workforce capacity or functional accessibility; physician counts omit full-time status, individual scopes of practice, or cross-county commuting, and facility availability does not guarantee timely services. In addition, a few counties were excluded from the analysis due to missing data. Third, the cross-sectional design limits causal inferences, and temporal trends were not examined. Because workforce and facility measures were assessed during 2023–2024, they may not fully reflect the historical healthcare environment that influenced current breast cancer outcomes, particularly mortality, which is affected by cancer latency, stage at diagnosis, treatment patterns, and survival over time. Therefore, the observed associations should be interpreted as contemporary county-level correlations rather than evidence that current provider or facility availability caused differences in breast cancer outcomes. Fourth, the BRFSS relies on self-report and may be subject to recall or social desirability bias. Available data was limited to mammography use among women aged 50–74 years, and screening estimates for women younger than 50 were not available. As such, our findings may not be generalizable to women outside the age range studied. The AHRF does not provide county-level data on the density of medical oncologists, and radiation oncology facility availability was not directly captured. Therefore, the treatment provider and facility variables used in this study should be interpreted as proxy measures of county-level cancer treatment capacity rather than comprehensive measures of all oncology services. However, the available provider-density measures demonstrated strong geographic correlation across counties, including surgeon density, radiation oncologist density, and internal medicine subspecialist physician density, indicating that physician workforce distribution tends to cluster geographically. Similar correlations were observed across facility-level indicators of healthcare infrastructure, including operating room and chemotherapy service availability. These findings support the use of the available workforce and facility measures as indicators of broader local treatment infrastructure, making substantial bias from the absence of direct measures unlikely, although residual misclassification remains possible. Finally, because the neighboring-county facility index was based on county contiguity, it may be subject to geographic edge effects for coastal or island counties and may not fully capture real-world travel patterns, cross-water access, or referral networks beyond immediately adjacent counties.

Nonetheless, our study has several notable strengths. To our knowledge, this is the first national county-level analysis to examine the interaction between physician workforce density and facility availability in relation to breast cancer outcomes. By leveraging multiple large-scale datasets and stratifying outcomes by facility presence, we were able to provide a more accurate understanding of how workforce capacity and infrastructure jointly affect screening, stage at diagnosis, and mortality. These findings not only reconcile prior conflicting evidence but also highlight the importance of considering both human and physical resources in shaping cancer outcomes. In doing so, our study provides a foundation for future research and offers effective insights to guide policy and investment strategies aimed at reducing disparities and improving breast cancer care.

## Conclusion

In this national county-level study of women aged 50–74 years, higher provider density was associated with more favorable breast cancer outcomes primarily in counties with available healthcare facilities. Higher screening provider density was associated with greater mammography use, but only in counties where mammography facilities were available. Similarly, higher treating provider density was linked to lower mortality rates, but only in counties with surgical and chemotherapy facilities. These findings underscore that healthcare infrastructure may condition the association between provider density and breast cancer outcomes, and that efforts to reduce disparities in breast cancer outcomes should invest in both.

## Data Availability

The data used in this study are publicly available from the Area Health Resource File, Behavioral Risk Factor Surveillance System, and Centers for Disease Control and Prevention. These data can be accessed through their respective public repositories.
