# Supplementary material for: Beyond the physician shortage: infrastructure as a rate-limiting step in breast cancer care
Source: Front Oncol. 2026 Jul 7;16:1826458. doi: 10.3389/fonc.2026.1826458 (PMC13384824; doi:10.3389/fonc.2026.1826458)

**Supplementary Methods**

Because the neighboring-county facility index was based on county contiguity, geographic edge effects may occur for counties located along coastlines, adjacent to large bodies of water, or on islands. Counties with fewer contiguous neighbors may have less stable neighboring-county index values because each adjacent county contributes more heavily to the calculated proportion. Counties with no identified contiguous neighboring counties were not assigned a neighboring-county facility index value and were treated as missing for analyses using this index. This approach avoids assigning an artificial value of 0 to counties without contiguous neighbors, which could incorrectly imply absence of regional facility access. However, this contiguity-based approach may not fully capture real-world access patterns, including travel across bridges, ferries, state boundaries, or referral networks that extend beyond immediately adjacent counties.

**Supplementary Sensitivity Analysis: Continuous Provider Density**

As a sensitivity analysis, provider density was modeled as a continuous variable and interacted with facility availability. This analysis was performed to assess whether the primary tertile-based findings were robust to modeling provider density continuously.

For breast cancer screening, screening provider density was not significantly associated with screening rate in counties without a mammography facility; each 100-provider increase per 100,000 women aged 50–75 years corresponded to an adjusted absolute difference of 0.018 percentage points in screening rate (p=0.664). In contrast, among counties with a mammography facility, higher screening provider density was significantly associated with higher screening rates, with each 100-provider increase corresponding to a 0.240-percentage-point higher screening rate (p<0.001).

For breast cancer mortality, treatment provider density was not significantly associated with mortality in counties without treatment facilities; each 100-provider increase corresponded to an adjusted absolute difference of −0.035 percentage points in mortality rate (p=0.350). In contrast, among counties with treatment facilities, higher treatment provider density was significantly associated with lower mortality, with each 100-provider increase corresponding to a 0.28-percentage-point lower mortality rate (p=0.023).

Together, these findings were consistent with the primary tertile-based analyses and suggest that the associations between provider density and breast cancer outcomes were stronger in counties with the relevant local facility infrastructure.

**Supplementary Sensitivity Analysis: Stratified by Facility Availability**

As a sensitivity analysis, we repeated the adjusted models after stratifying counties by facility availability, using low provider density as the reference group within each facility stratum.

For breast cancer screening, provider density was not significantly associated with screening rates among counties without mammography facilities. Compared with counties with low provider density, counties with moderate provider density had a nonsignificant increase in screening rate (β = 0.348; 95% CI, -0.181 to 0.877; p = 0.197), as did counties with high provider density (β = 0.316; 95% CI, -0.289 to 1.002; p = 0.279). In contrast, among counties with mammography facilities, high provider density was significantly associated with higher screening rates compared with low provider density (β = 0.529; 95% CI, 0.128 to 1.186; p = 0.015), while moderate provider density was not associated with increased screening rates (β = 0.017; 95% CI, -0.774 to 0.741; p = 0.966).

For breast cancer mortality, provider density was again not significantly associated with mortality among counties without treatment facilities. Compared with counties with low provider density, moderate provider density was associated with a nonsignificant decrease in mortality (β = -0.529; 95% CI, -1.116 to 0.142; p = 0.129), and high provider density was also not significant (β = -0.219; 95% CI, -0.696 to 0.258; p = 0.370). Among counties with treatment facilities, however, both moderate and high provider density were significantly associated with lower breast cancer mortality compared with low provider density. Moderate provider density was associated with lower mortality (β = -0.852; 95% CI, -1.713 to -0.108; p = 0.026), as was high provider density (β = -1.241; 95% CI, -2.187 to -0.394; p = 0.005).

Overall, these stratified results were consistent with the primary interaction analysis and support the interpretation that provider availability is associated with improved breast cancer outcomes primarily in counties where the relevant facility infrastructure is also present.

**Supplementary Figure 1. Conceptual model representing the association between county-level provider density, facility availability, and breast cancer outcomes.** Demographic and health-related behavioral county-level characteristics were conceptualized as potential confounders of the association between provider density and breast cancer outcomes and were included as adjustment covariates. Provider density was modeled as the primary exposure of interest, while facility availability was evaluated as an effect modifier of the association between provider density and screening, late-stage diagnosis, and mortality. The blue arrow represents the primary adjusted association of interest, and the dashed red arrow represents hypothesized effect modification by local facility availability.


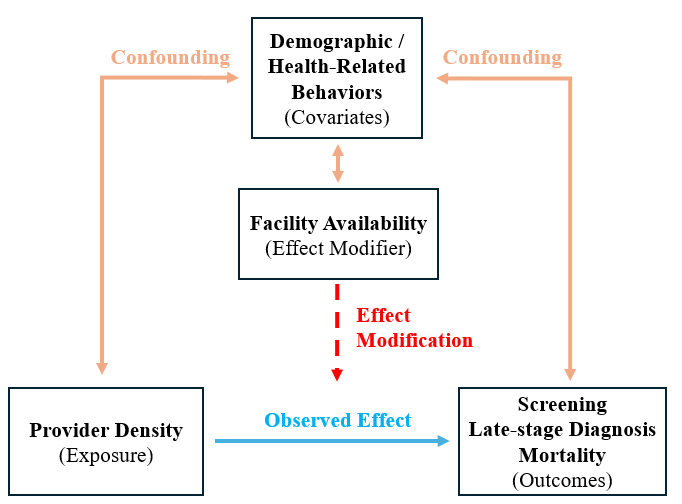

Supplement: Supplementary file 1 [file DataSheet1.docx]
